# Differences in Technical Performance of Players From ‘The Big Five’ European Football Leagues in the UEFA Champions League

**DOI:** 10.3389/fpsyg.2019.02738

**Published:** 2019-12-06

**Authors:** Qing Yi, Ryan Groom, Chen Dai, Hongyou Liu, Miguel Ángel Gómez Ruano

**Affiliations:** ^1^School of Physical Education and Sport Training, Shanghai University of Sport, Shanghai, China; ^2^Key Laboratory of Diagnosis and Analysis of Skills and Tactics in Sports, Shanghai University of Sport, Shanghai, China; ^3^Facultad de Ciencias de la Actividad Física y del Deporte (INEF), Universidad Politécnica de Madrid, Madrid, Spain; ^4^Department of Sport and Exercise Sciences, Manchester Metropolitan University, Manchester, United Kingdom; ^5^College of Physical Education, Hunan Normal University, Changsha, China; ^6^School of Physical Education and Sports Science, South China Normal University, Guangzhou, China; ^7^National Demonstration Center for Experimental Sports Science Education, South China Normal University, Guangzhou, China

**Keywords:** performance analysis, match analysis, player profiles, European soccer leagues, playing style

## Abstract

The current study aimed to identify the differences in technical performance between players from clubs of Bundesliga (Germany), La Liga (Spain), Ligue 1 (France), Premier League (England) and Serie A (Italy) when competing in the matches of the UEFA Champions League. Technical performance-related match data of 1,291 players from 1,125 matches (9,799 observations) of the UEFA Champions League (seasons 2009/2010–2017/2018) were collected and analysed. The generalised mixed linear modelling was employed taking the names of the league as the independent variable to predict the count number of 20 technical performance-related match actions and events performed by players belonging to different leagues. The non-clinical magnitude-based inference was used to evaluate the uncertainty in the true effects of the predictor. Results showed that differences in the technical performances between players from La Liga, Premier League and Ligue 1 were all trivial. Bundesliga players made higher numbers of shots than players from La Liga, Premier League and Serie A and achieved more long balls than players from Ligue 1. Serie A players achieved lower numbers of ball touches, passes and lower pass accuracy per match than players from any of the other four leagues. In addition, players from Serie A performed a higher number of long balls per match than Ligue 1 players and lower number of dribbles per match than Premier League players. Non-significant differences in other variables related to passing and organising and all variables related to defending were identified in players between the five leagues. The identified differences in technical performance among leagues could provide a more thorough understanding for practitioners working within the fields of talent identification, player development, player recruitment, coaching and match preparation.

## Introduction

Spanish La Liga, English Premier League, Italian Serie A, German Bundesliga and French Ligue 1 are the top five ranked professional football leagues in the Union of European Football Associations (UEFA)^1^ and have been recognised to be the most successful football leagues in the world (Lago-Peñas et al., 2016). The success of ‘the big five’ leagues from a financial perspective has enabled teams to attract top-level players from all over the world (Littlewood et al., 2011; Oberstone, 2011).

Each of these five leagues is characterised by an idiosyncratic match-play style due to the cultural, historical and social differences existing among countries (Sarmento et al., 2013; Sapp et al., 2018). Thus, players or teams from the same league are often characterised by a specific archetype (Sarmento et al., 2013). Teams from the English Premier League, have tended to be characterised by a direct style with special emphasis upon its importance of the players’ physical attributes (e.g., strength, speed, power etc.) (Dellal et al., 2011). The English Premier League is therefore considered as the most aggressive league (Sapp et al., 2018). Italian teams are often characterised by a rigid tactical requirement for defensive organisation (Foot, 2006). Spanish teams are often considered to prioritise ball possession and players’ individual technical ability to control the game (Crolley et al., 2000) whilst the German and French Leagues are often considered to fall somewhere between the English tendency for physically able players and the Italian desire for defencive team organisation (Oberstone, 2011; Sapp et al., 2018).

Nevertheless, the playing style observed within each of the leagues has evolved over years (Bloomfield et al., 2005), as a result of the increasing frequency of player and coach labour migration between leagues in modern football (Frick, 2007; Littlewood et al., 2011). Even so, differences between the major European football leagues have been identified from a number of perspectives. For example, a qualitative analysis from Sarmento et al. (2013) demonstrated that the differences exist between La Liga (Spain), the Premier League (England) and Serie A (Italy) regarding culture, preferred strategy and tactics, players’ characteristics and coaches’ philosophies. Moreover, there have been a number of comparative studies conducted to examine the differences between major European leagues that have focussed upon: anthropometric data (Bloomfield et al., 2005), technical and tactical performance (Dellal et al., 2011; Oberstone, 2011; Alberti et al., 2013; Collet, 2013; Lago-Peñas et al., 2016; Sapp et al., 2018), physical performance (Dellal et al., 2011), injury pattern (Waldén et al., 2005), player recruitment (Frick, 2007; Littlewood et al., 2011) and competitive balance (Brandes and Franck, 2007; Pawlowski et al., 2010). Even though anecdotal attention has been paid to the differences in technical and tactical performance in different leagues, and the effects of situational variables and playing position were also considered, but either limited performance-related variables or a limited number of leagues have previously been analysed. Besides, those comparisons were not properly accounted for the effects of situational variables as players’ match performances are varied in different competing contexts (Gómez et al., 2013). Therefore, there is an important empirical and practical gap in our understanding of the differences in match-play between major European leagues.

Methodologically, one of the major obstacles that affects our ability to conduct an objective and accurate assessment of player’s match performance is the comparability of match data across leagues due to the inconsistency in data collection and analysis systems (Barros et al., 2007; Di Salvo et al., 2009). Therefore, match data collected from the UEFA Champions League (a cross-league competition), which is standardised for all teams, is an ideal source of data for scientific comparison. In addition, from a data analysis perspective, a methodology is needed to address appropriately the repeated-measured data of players who played in multiple matches (Yi et al., 2018, 2019), which was failed to be considered in previous research (Dellal et al., 2011; Oberstone, 2011; Alberti et al., 2013; Lago-Peñas et al., 2016; Sapp et al., 2018). The generalised mixed linear modelling used in the current study has been proved as a valid method for this issue, and the situational effects can also be controlled by this modelling to identify the similarities and differences between leagues in a more objective way (Yi et al., 2018, 2019).

Consequently, the current study aims to quantify the differences of technical performance of players from five major European leagues (Spanish La Liga, English Premier League, Italian Serie A, German Bundesliga and French Ligue 1) in the UEFA Champions League from season 2009/2010 to 2017/2018. The large sample size allows us to account for the effects of situational variables (game location: home, away and neutral, team and opponent strength: end-of-season UEFA club coefficient, match type: group stage and knockout stage and game result: win, draw and lose) and the effect of players’ playing position (central defender, full back, central midfielder, wide midfielder and forward). Using generalised mixed linear modelling, allows us to demonstrate the similarities and differences between leagues with an increased degree of confidence. What is more, an added random effect of player names in the modelling, could process properly the repeated-measured data of a player participating in various matches.

## Materials and Methods

### Data Resource and Reliability

Technical performance-related match data of players in all the 1,125 games throughout nine seasons from 2009–2010 to 2017–2018 in the UEFA Champions League were collected manually from a public-accessed football statistic website named ‘whoscored.com’^2^. The original data of this website is provided by OPTA Sportsdata company, whose tracking system (OPTA Client System) has been tested by Liu et al. (2013) to have a high level of reliability (*Kappa* values > 0.90). The current study maintains the anonymity of the players according to European data protection law, and follows the research ethics standard set out by the local university.

### Sample and Variables

The dataset consisted of 9,799 full-match observations of 1,291 players from Spanish La Liga (*n* = 2,597 observations), English Premier League (*n* = 2,303), Italian Serie A (*n* = 1,522), German Bundesliga (*n* = 2,021) and French Ligue 1 (*n* = 1,356) played in the UEFA Champions League during the past nine seasons (2009/2010–2017/2018). Only the data of match observations of outfield players who played the entire match were included. Moreover, due to the differences in technical variables between outfield players and goalkeepers, technical performance-related match data of goalkeepers were excluded from the sample.

After the screening based on the availability of data and the review of previous literature (Lago-Peñas et al., 2010; Castellano et al., 2012; Liu et al., 2015, 2016; Yi et al., 2018), 20 technical performance-related match actions and events grouped into four groups were selected as the dependent variables in the analysis (see Table 1).

**TABLE 1:** Selected technical performance-related match events and actions.

**Groups**	**Event or action: operational definition**
Variables related to goal scoring	Shot: an attempt to score a goal, made with any (legal) part of the body, either on or off target Shot on target: an attempt to goal which required intervention to stop it going in or resulted in a goal/shot which would go in without being diverted
Variables related to passing and organising	Touch: a sum of count values of all actions and events where a player touches the ball Pass: an intentional played ball from one player to another Pass accuracy (%): successful passes as a proportion of total passes Key pass: the final pass or cross leading to the recipient of the ball having an attempt at goal without scoring Cross: any ball sent into the opposition team’s area from a wide position Long ball: an attempted pass of 25 yards or more Through ball: a pass that split the last line of defence and plays the teammate through on goal Aerial won: two players competing for a ball in the air, for it to be an aerial duel both players must jump and challenge each other in the air and have both feet off the ground. The player who wins the duel gets the *Aerial won*, and the player who does not gets an *Aerial lost* Dribble: a dribble is an attempt by a player to beat an opponent in possession of the ball. OPTA also log attempted dribbles where the player overruns the ball Offside: awarded to the player deemed to be in an offside position where a free kick is awarded. If two or more players are in an offside position when the pass is played, the player considered to be most active and trying to play the ball is given offside Fouled: where a player is fouled by an opponent Dispossessed: when a player is tackled without attempting to dribble past his opponent
Variables related to defending	Tackle: the action of gaining possession from an opposition player who is in possession of the ball Interception: a player intercepts a pass with some movement or reading of the play Clearance: attempt made by a player to get the ball out of the danger zone, when there is pressure (from opponents) on him to clear the ball Blocked shot: a defensive block, blocking a shot going on target. This must be awarded to the player who blocks the shot Foul: any infringement that is penalised as foul play by a referee Yellow card: where a player was shown a yellow card by the referee for reasons of foul, persistent infringement, hand ball, dangerous play, etc.

### Procedure and Statistical Analysis

Generalised mixed linear modelling was achieved by *Proc Glimmix* in the University Edition of Statistical Analysis System (version SAS Studio 3.6). The fixed effects estimated the effects of league, game location, team and opponent strength, match type and game result, as well as playing position. Player identity was used as the random effect to interpret the repeated-measure data of players from multiple matches. Separate Poisson regressions were run in the model taking the value of each of the 20 technical performance-related variables as the dependent variable.

League, game location, match type, game result and playing position were all included as nominal predictor variables in the model. League was with five levels (Spanish La Liga, English Premier League, Italian Serie A, German Bundesliga and French Ligue 1), game location was with three levels (home, away and neutral), match type was with two levels (matches of group stage and knock-out stage), game result was with three levels (win, draw and loss), and playing position was with five levels (CD, FB, CM, WM and FW). The effect of team and opponent strength was estimated by including the difference in the log of the end-of-season UEFA club coefficient as a predictor (Yi et al., 2018).

Uncertainty in the true effects of the predictors was evaluated using non-clinical magnitude-based inference that referenced *Bayesian* with a dispersed uniform prior which was implemented in the spreadsheet accompanying the package of materials for generalised mixed modelling with SAS Studio (Hopkins, 2016). Estimated magnitudes of difference in means and their 90% confidence limits were presented in standardised units, and were evaluated qualitatively with the following scale: trivial, 0–0.2; small, 0.2–0.6; moderate, 0.6–1.2; large, 1.2–2.0; and very large, >2.0 (Hopkins et al., 2009). The likelihood of the effects to be clear was considered qualitatively as follows: <0.5%, most unlikely; 0.5–5%, very unlikely; 5–25%, unlikely; 25–75%, possibly; 75–95%, likely; 95–99.5%, very likely; and >99.5%, most likely (Hopkins et al., 2009).

## Results

**FIGURE 1:**
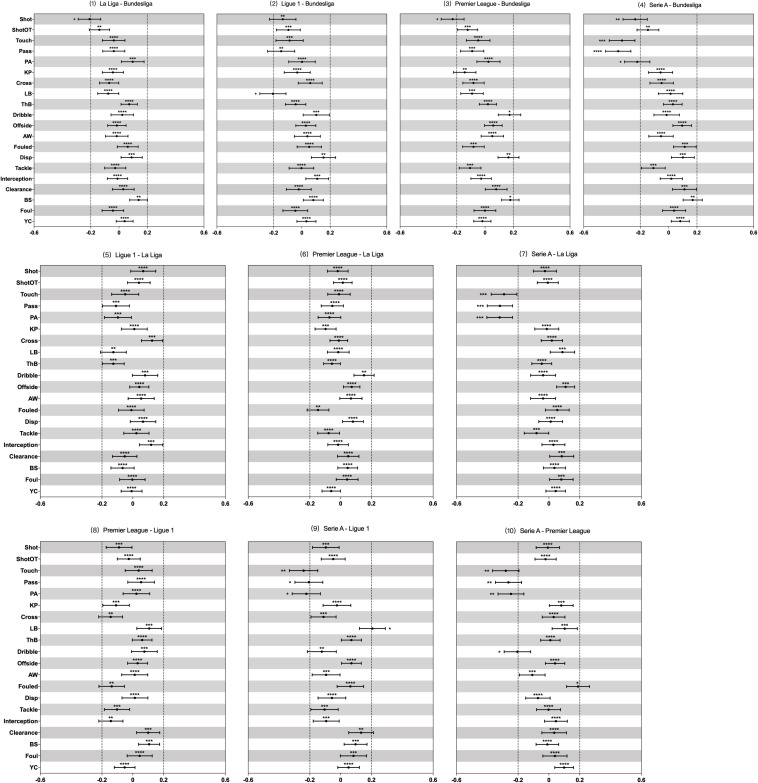
Standardised effects of differences in mean count of each match action or event between five UEFA leagues estimated from the generalised mixed linear modelling. Bars are 90% confidence intervals. Asterisks indicate the likelihood for the magnitude of the true effect as follows: ^∗^possible; ^∗∗^likely; ^∗∗∗^very likely; ^****^most likely. Asterisks located in the area between –0.2 and 0.2 denote for trivial differences. ShotOT, shot on target; PA, pass accuracy in %; KP, key pass; LB, long ball; ThB, through ball; AW, aerial won; Disp, dispossessed; BS, blocked shot; YC, yellow card.

Descriptive statistics (mean ± SD) of the 20 technical performance-related match variables for players from five European football leagues estimated by the generalised mixed linear model controlling the effects of playing position, game location, match type, game result and team and opposition strength are presented in Table 2. The estimated true effects (effect size ± 90% confidence interval) of differences within pairwise comparisons between any two of these five leagues are shown in the Figure 1.

**TABLE 2:** Descriptive statistics of technical match performance of players from big five UEFA leagues estimated from the generalised mixed linear model (*n* denotes the number of match observations, results are mean ± between-player standard deviation, units are counts, except for pass accuracy).

**Variable**	**Bundesliga (*n* = 2,021)**	**La Liga (*n* = 2,597)**	**Ligue 1 (*n* = 1,356)**	**Premier League (*n* = 2,303)**	**Serie A (*n* = 1,522)**
Shot	1.19 ± 1.56	0.95 ± 1.40	1.03 ± 1.45	0.93 ± 1.38	0.92 ± 1.38
Shot on target	0.38 ± 0.95	0.30 ± 0.91	0.33 ± 0.92	0.31 ± 0.91	0.30 ± 0.90
Touch	62.33 ± 20.96	61.57 ± 20.79	60.57 ± 20.55	61.34 ± 20.73	55.90 ± 19.45
Pass	45.55 ± 19.62	44.86 ± 19.41	42.85 ± 18.82	43.85 ± 19.12	39.25 ± 17.74
Pass accuracy (%)	81.16 ± 9.59	82.09 ± 9.66	81.17 ± 9.59	81.39 ± 9.61	79.06 ± 9.43
Key pass	0.89 ± 1.39	0.84 ± 1.36	0.85 ± 1.37	0.74 ± 1.29	0.83 ± 1.35
Cross	1.16 ± 2.66	1.04 ± 2.50	1.28 ± 2.82	1.02 ± 2.47	1.08 ± 2.54
Long ball	3.60 ± 3.32	3.38 ± 3.19	3.04 ± 3.00	3.34 ± 3.17	3.64 ± 3.34
Through ball	0.12 ± 0.99	0.15 ± 0.95	0.11 ± 1.02	0.13 ± 0.98	0.13 ± 0.97
Dribble	0.67 ± 1.40	0.70 ± 1.42	0.78 ± 1.50	0.87 ± 1.58	0.66 ± 1.39
Offside	0.11 ± 0.89	0.11 ± 0.90	0.12 ± 0.88	0.13 ± 0.87	0.14 ± 0.86
Aerial won	1.06 ± 1.66	1.04 ± 1.65	1.11 ± 1.70	1.13 ± 1.71	0.99 ± 1.61
Fouled	1.14 ± 1.41	1.22 ± 1.45	1.21 ± 1.44	1.05 ± 1.36	1.29 ± 1.48
Dispossessed	0.80 ± 1.30	0.89 ± 1.37	0.97 ± 1.42	0.98 ± 1.43	0.91 ± 1.38
Tackle	2.03 ± 1.89	1.98 ± 1.87	2.02 ± 1.89	1.86 ± 1.81	1.85 ± 1.81
Interception	1.55 ± 1.69	1.53 ± 1.68	1.72 ± 1.77	1.51 ± 1.67	1.58 ± 1.70
Clearance	1.48 ± 2.17	1.54 ± 2.20	1.45 ± 2.14	1.63 ± 2.27	1.69 ± 2.32
Blocked shot	0.20 ± 0.92	0.27 ± 0.93	0.24 ± 0.92	0.30 ± 0.94	0.29 ± 0.94
Foul	1.34 ± 1.47	1.29 ± 1.44	1.29 ± 1.44	1.34 ± 1.47	1.39 ± 1.49
Yellow card	0.17 ± 0.71	0.19 ± 0.70	0.18 ± 0.70	0.16 ± 0.71	0.20 ± 0.70

Regarding the variables related to goal scoring, the number of shots from players of German Bundesliga was clearly higher than that of Spanish La Liga, English Premier League and Italian Serie A, while no substantial differences were observed among shots attempted by players of other four leagues. There were no clear differences ascertained in shot on target between players from all five leagues. No substantial differences were detected between players from Spanish La Liga, English Premier League and German Bundesliga when considering the variables related to passing and organising. However, comparing to the players from other four leagues, players of Italian Serie A performed a lower number of touches, passes and achieved lower pass accuracy. Additionally, players from German Bundesliga and Italian Serie A achieved more long balls than players from French Ligue 1. Players from English Premier League had greater number of dribbles compared with those players from Italian Serie A. Other pairwised comparisons across five leagues regarding variables related to passing and organising did not reveal meaningful differences. Furthermore, trivial differences were also observed between players from all five leagues in all variables related to defending.

## Discussion

The current study quantified the differences in technical match performance between players from the ‘major five’ European football leagues in the UEFA Champions League. The data set presented here was based on one of the largest samples of data published to date (nine seasons). Moreover, our research approach allows us to control the situational effects for the identification of similarities and differences, and the UEFA Champions League and domestic leagues are characterised by specific features. Therefore, the findings from the current study are partly differ from prior studies due to the different methodologies used and the data sources. The current study demonstrated that the differences in players’ technical performance between five leagues were smaller than we expected from the stereotypical perspective often portrayed within the media. Differences were mainly focussed on the variables related to goal scoring, passing and organising, whereas differences in variables related to defending were minimal.

Our study suggests that players from La Liga (Spain), the Premier League (England) and Ligue 1 (France) performed similar match actions and no substantial differences were observed across all technical variables. This finding differ from past research that has suggested that players from La Liga (Spain) performed a more elaborated and skilled playing style while players from the Premier League (England) prefer the direct playing style, they performed relatively more tackles and aerial duels and covered greater distances in sprinting (Dellal et al., 2011; Oberstone, 2011; Sarmento et al., 2013). In addition, a previous research from Oberstone (2011) reported that significant differences existed between La Liga (Spain) and the Premier League (England) in variables related to goal scoring and defending. Ligue 1 (France) has received comparatively less attention in existing studies and therefore offers a novel area of exploration. Our findings revealed that when competing in the UEFA Champions League, players from Ligue 1 (France) shared a similar technical performance to players from La Liga (Spain) and the Premier League (England). This finding may reveal a trend that the differences in technical performance among these three leagues has decrease over time, potentially due to increasing levels of player and coach migration (Maguire and Stead, 1998; Maguire and Pearton, 2000; Littlewood et al., 2011; Sarmento et al., 2013). Hence, the teams’ style of play and players’ technical characteristics had evolved and were assimilated (Maguire and Stead, 1998; Maguire and Pearton, 2000).

Meaningful differences in technical performance between players from the Bundesliga (Germany), La Liga (Spain) and the Premier League (England) were only observed in the number of shots per game. Players from the Bundesliga (Germany) performed the most shots per game, while the differences in shots on target were minimal among these three leagues. This is partly in contrast to the findings of Oberstone (2011), who illustrated that La Liga (Spain) has a higher percentage of shot on target than the Premier League (England). Considering the technical differences between players from the Bundesliga (Germany) and Ligue 1 (France), we only found substantial difference in the number of long balls. Players from the Bundesliga (Germany) performed more long balls than players from Ligue 1 (France). Interestingly, the number of long balls from players from Serie A (Italy) was also substantially higher than those of Ligue 1 (France). Long balls have previously been related to the direct playing style (Fernandez-Navarro et al., 2016; Lago-Peñas et al., 2017). This finding may indicate that direct play is more prevalent in the Bundesliga (Germany) and Serie A (Italy) using long aerial passes more frequently than in the Premier League (England), La Liga (Spain) and Ligue 1 (France).

A prior study from Oberstone (2011) compared the technical performance of players from the Premier League (England), Serie A (Italy) and La Liga (Spain) based on a database of domestic leagues, it was reported that Serie A (Italy) is a league with better passing compared with the Premier League (England) and La Liga (Spain). However, the current study demonstrated that players from Serie A (Italy) showed comparatively greater technical difference during the comparisons between five leagues, especially in the aspect of passing and organising. They achieved the lowest ball touches, passes and pass accuracy comparing to their counterparts from the other four leagues (e.g., the Bundesliga, La Liga, Ligue 1 and the Premier League). This result may indicate that there is no passing superiority for players from Serie A (Italy) when competing in the international competition against the best teams from other leagues. We also found that players from Serie A (Italy) achieved the highest number of long passes, although the meaningful differences only observed in the comparison with players from Ligue 1 (France). This finding combined with their abovementioned weakness support the notion that Italian teams prefer to play counter-attack and are more focussed on the tactical-defensive aspects in the UEFA Champions league. Therefore, the low number of touch and pass were obtained. In addition, long pass is a crucial element during the counter-attack process, the high frequencies of occurrence in long balls could influence the accuracy of pass (Liu et al., 2016).

Given the focus on the perspective of attacking. The popular assumption is that La Liga (Spain) is primarily attacking and trying to control the match with high ball possession, while Serie A (Italy) has been characterised by its rigorous defensive tactics and relatively less developed and worse prepared in offencive aspect (Sarmento et al., 2013). Conversely, the results of the present study showed that limited differences in attacking performances between players from five leagues. The only substantial difference was detected in dribble between players of the Premier League (England) and Serie A (Italy). Dribble is a performance indicator that could reflect the degree of freedom of players during match play (Liu et al., 2016). In the Premier League (England), tactical and strategic aspects have not tended to have been considered with the same importance as in other leagues, while Italian Serie A has a special emphasis on its collective execution in the defensive organisation and plays a conservative game (Sarmento et al., 2013). Therefore, players from English Premier League are given more freedom to perform technical skills (dribble) during the attacking process, mainly due to direct styles of play with more counterattacks (Fernandez-Navarro et al., 2016).

Previously it has been suggested that teams in Italian Serie A are better in defending performance than other European teams (Sarmento et al., 2013). However, one noteworthy finding in this study is that players from all five leagues showed no substantial differences in defensive variables. This may reveal a trend that teams are increasingly aware of the importance of a solid defence for winning a game, trying to keep a good balance between attacking and defending during match play. The introduction of a considerable number of foreign players and coaches may once again account for that why the differences that we expected did not show in the current study. The foreign players that grown up in different youth academy systems have been instilled different conceptions about attacking and defending (Maguire and Stead, 1998; Maguire and Pearton, 2000), there will be an interplay between players from different countries when they play in the same team. Moreover, the migration of players and coaches between leagues may reduce the differences in players’ physical abilities and teams’ playing style between different leagues (Bloomfield et al., 2005; Sarmento et al., 2013). Therefore, these two factors may explain that the defending variables are no longer the important indicators to differentiate the five leagues. In addition, the evolution of tactics and strategies may also play a role. The diversity of tactics and strategies in modern football enable teams to find a defensive tactic that could be suitable for their playing style in defence.

## Conclusion

This study identified both similarities and disparities in various aspects of players’ technical performance across major five European football leagues in the UEFA Champion League from a long-term perspective. Players from the Bundesliga (Germany) were more advantageous than other leagues for creating scoring opportunities. Serie A (Italy) players did not show their advantage in defending performance but showed the worst performance in passing and organising. Players from La Liga (Spain) and the Premier League (England) have a balance performance in all aspects. However, the superiority of La Liga players in passing and organising was not detected and Premier League players are no longer playing the ‘aerial game’ as we used to think from the stereotypical perspective. Little attention has been paid on the comparison of players from Ligue 1 (France) with other leagues, while our data suggested that their technical performance were similar to players from La Liga (Spain) and the Premier League (England), but they prefer short pass during the attacking process. These technical differences largely driven by the differences in culture, playing style, players’ characteristics and coach’s philosophy. Nevertheless, the evolution of tactics and strategies, the migration of players and coaches and the different systems in youth academy may also play a role.

### Practical Applications

The differences in technical performance for players from each league have been identified, thus the technical profiles could be created, which can provide a more thorough understanding for stakeholders in talent identification, player development, player recruitment, coaching and the match preparation when against with foreign teams. However, limitations of this study should be noted. This study only compared the technical players’ performance from the best teams of each league played in a European competition rather than the whole teams of a domestic league. Therefore, the players’ characteristics from a specific league identified in this study cannot represent the general characteristics of that entire league. This is one of the main causes that the disparities of findings between the current study and previous studies. Moreover, only the statistics of technical match actions and events were analysed, while the information of tactical behaviours was failed to consider due to the availability of match data. This may restrict the understanding of players’ technical and tactical match performance. Lastly, further studies should account for validity and reliability of the sample used and key performance indicators in soccer as was previously described in basketball (Pérez-Ferreirós et al., 2019). This approach will be extremely useful when studying the minimum (reliable and valid) number of matches or observations from soccer teams and players, respectively.

## Data Availability Statement

The datasets generated for this study are available on request to the corresponding author.

## Ethics Statement

The studies involving human participants were reviewed and approved by the Ethics Committee of the Polytechnic University of Madrid. Written informed consent for participation was not required for this study in accordance with the national legislation and the institutional requirements.

## Author Contributions

QY participated in the study design, data collection, statistical analysis, data interpretation, writing, and revision of the manuscript. RG participated in the revision and proof check of the manuscript. CD participated in the data collection. HL and MG participated in the study design, statistical analysis, data interpretation, and revision of the manuscript.

## Conflict of Interest

The authors declare that the research was conducted in the absence of any commercial or financial relationships that could be construed as a potential conflict of interest.

## Footnotes

1 http://www.uefa.com

2 https://www.whoscored.com

## References

[B1] Alberti G. Iaia F. M. Arcelli E. Cavaggioni L. Rampinini E. (2013). Goal scoring patterns in major European soccer leagues. *Sport Sci. Health* 9 151–153. 10.1007/s11332-013-0154-9

[B2] Barros R. M. Misuta M. S. Menezes R. P. Figueroa P. J. Moura F. A. Cunha S. A. (2007). Analysis of the distances covered by first division Brazilian soccer players obtained with an automatic tracking method. *J. Sports Sci. Med.* 6:233. 24149334 PMC3786245

[B3] Bloomfield J. Polman R. Butterly R. O’Donoghue P. (2005). Analysis of age, stature, body mass, BMI and quality of elite soccer players from 4 European Leagues. *J. Sports Med. Phys. Fitness* 45 58–67. 16208292

[B4] Brandes L. Franck E. (2007). Who made who? An empirical analysis of competitive balance in European soccer leagues. *East. Econ. J.* 33 379–403. 10.1057/eej.2007.32

[B5] Castellano J. Casamichana D. Lago C. (2012). The use of match statistics that discriminate between successful and unsuccessful soccer teams. *J. Hum. Kinet.* 31 137–147. 10.2478/v10078-012-0015-7 23487020 PMC3588662

[B6] Collet C. (2013). The possession game? A comparative analysis of ball retention and team success in European and international football, 2007–2010. *J. Sports Sci.* 31 123–136. 10.1080/02640414.2012.727455 23067001

[B7] Crolley L. Hand D. Jeutter R. (2000). Playing the identity card: stereotypes in European football. *J. Soccer Soc.* 1 107–128. 10.1080/14660970008721267

[B8] Dellal A. Chamari K. Wong D. P. Ahmaidi S. Keller D. Barros R. (2011). Comparison of physical and technical performance in European soccer match-play: FA premier league and La Liga. *Eur. J. Sport Sci.* 11 51–59. 10.1080/17461391.2010.481334

[B9] Di Salvo V. Gregson W. Atkinson G. Tordoff P. Drust B. (2009). Analysis of high intensity activity in premier league soccer. *Int. J. Sports Med.* 30 205–212. 10.1055/s-0028-1105950 19214939

[B10] Fernandez-Navarro J. Fradua L. Zubillaga A. Ford P. R. McRobert A. P. (2016). Attacking and defensive styles of play in soccer: analysis of Spanish and english elite teams. *J. Sports Sci.* 34 2195–2204. 10.1080/02640414.2016.1169309 27052355

[B11] Foot J. (2006). *Calcio. A History of Italian Football.* New York, NY: Harper Perennial.

[B12] Frick B. (2007). The football players’ labour market: empirical evidence from the major European leagues. *Scott. J. Polit. Econ.* 54 422–446. 10.1111/j.1467-9485.2007.00423.x

[B13] Gómez M. Lago C. Pollard R. (2013). “Situational variables,” in *Routledge Handbook of Sports Performance Analysis*, eds McGarry T. O’Donoghue P. Sampaio J. (London: Routledge), 259–269.

[B14] Hopkins W. (2016). SAS (and R) for mixed models. Spreadsheet: process poisson and logistic repeated measures. *Sport Sci.* 20:3.

[B15] Hopkins W. Marshall S. Batterham A. Hanin J. (2009). Progressive statistics for studies in sports medicine and exercise science. *Med. Sci. Sports Exerc.* 41 3–13. 10.1249/mss.0b013e31818cb278 19092709

[B16] Lago-Peñas C. Gómez-Ruano M. Megías-Navarro D. Pollard R. (2016). Home advantage in football: examining the effect of scoring first on match outcome in the five major European leagues. *Int. J. Perform. Anal. Sport* 16 411–421. 10.1080/24748668.2016.11868897

[B17] Lago-Peñas C. Gómez-Ruano M. Yang G. (2017). Styles of play in professional soccer: an approach of the Chinese soccer super league. *Int. J. Perform. Anal. Sport* 17 1073–1084. 10.1080/24748668.2018.1431857

[B18] Lago-Peñas C. Lago-Ballesteros J. Dellal A. Gómez M. (2010). Game-related statistics that discriminated winning, drawing and losing teams from the Spanish soccer league. *J. Sports Sci. Med.* 9 288–293. 24149698 PMC3761743

[B19] Littlewood M. Mullen C. Richardson D. (2011). Football labour migration: an examination of the player recruitment strategies of the ‘big five’European football leagues 2004–5 to 2008–9. *Soccer Soc.* 12 788–805. 10.1080/14660970.2011.609680

[B20] Liu H. Gómez M.-A. Gonçalves B. Sampaio J. (2016). Technical performance and match-to-match variation in elite football teams. *J. Sports Sci.* 34 509–518. 10.1080/02640414.2015.1117121 26613399

[B21] Liu H. Hopkins W. Gómez A. M. Molinuevo S. J. (2013). Inter-operator reliability of live football match statistics from OPTA Sportsdata. *Int. J. Perform. Anal. Sport* 13 803–821. 10.1080/24748668.2013.11868690

[B22] Liu H. Yi Q. Giménez J.-V. Gómez M.-A. Lago-Peñas C. (2015). Performance profiles of football teams in the UEFA champions league considering situational efficiency. *Int. J. Perform. Anal. Sport* 15 371–390. 10.1080/24748668.2015.11868799

[B23] Maguire J. Pearton R. (2000). The impact of elite labour migration on the identification, selection and development of European soccer players. *J. Sports Sci.* 18 759–769. 10.1080/02640410050120131 11043901

[B24] Maguire J. Stead D. (1998). Border crossings: soccer labour migration and the European Union. *Int. Rev. Sociol. Sport* 33 59–73. 10.1177/101269098033001005 11043901

[B25] Oberstone J. (2011). Comparing team performance of the english premier league, Serie A, and La Liga for the 2008-2009 season. *J. Quant. Anal. Sports* 7 1–18.

[B26] Pawlowski T. Breuer C. Hovemann A. (2010). Top clubs’ performance and the competitive situation in European domestic football competitions. *J. Sports Econ.* 11 186–202. 10.1177/1527002510363100

[B27] Pérez-Ferreirós A. Kalén A. Gómez M. Á Rey E. (2019). Reliability of Teams’ game-related statistics in basketball: number of games required and minimal detectable change. *Res. Q. Exerc. Sport* 90 297–306. 10.1080/02701367.2019.1597243 31046653

[B28] Sapp R. M. Spangenburg E. E. Hagberg J. M. (2018). Trends in aggressive play and refereeing among the top five European soccer leagues. *J. Sports Sci.* 36 1346–1354. 10.1080/02640414.2017.1377911 28895469

[B29] Sarmento H. Pereira A. Matos N. Campaniço J. Anguera T. M. Leitão J. (2013). English premier league, spaiǹs La Liga and Italýs seriés A–What’s different? *Int. J. Perform. Anal. Sport* 13 773–789.

[B30] Waldén M. Hägglund M. Ekstrand J. (2005). UEFA Champions League study: a prospective study of injuries in professional football during the 2001–2002 season. *Br. J. Sports Med.* 39 542–546. 10.1136/bjsm.2004.014571 16046340 PMC1725291

[B31] Yi Q. Gómez M. A. Wang L. Huang G. Zhang H. Liu H. J. (2019). Technical and physical match performance of teams in the 2018 FIFA World cup: effects of two different playing styles. *J. Sports Sci.* 37 2569–2577. 10.1080/02640414.2019.1648120 31354060

[B32] Yi Q. Jia H. Liu H. Gómez M. Á (2018). Technical demands of different playing positions in the UEFA Champions league. *Int. J. Perform. Anal. Sport* 18 926–937. 10.1080/24748668.2018.1528524

